# A case of hypertensive emergency in a patient who had a history of very low birth weight

**DOI:** 10.1007/s13730-026-01119-0

**Published:** 2026-04-18

**Authors:** Yoichi Kadoh, Jun Yoshino, Tomohiro Oka, Fumika Kamei, Ai Uchida, Maki Hanada, Mamiko Nagase, Daisuke Niino, Takeshi Kanda

**Affiliations:** 1 Division of Nephrology, Department of Internal Medicine, Faculty of Medicine, Shimane University, 89-1 Enya-Cho, Izumo, Shimane 693-8501 Japan; 2 The Center for Integrated Kidney Research and Advance (IKRA), Shimane University, Izumo, Shimane Japan; 3 Department of Functional Pathology, Faculty of Medicine, Shimane University, Izumo, Shimane Japan

**Keywords:** Hypertensive emergency, Hypertension, Low birth weight, Acute kidney injury

## Abstract

Low birth weight (LBW) is a risk factor for development of hypertension and chronic kidney disease in adulthood. Here, we report the case of a forty-year-old man with a history of very low birth weight (VLBW) (1300 g) who had hypertensive emergency (233/142 mmHg), acute kidney injury (AKI) (serum creatinine, 8.47 mg/dL), elevated plasma renin activity (102 ng/mL/h), and posterior reversible encephalopathy syndrome. He received temporary hemodialysis but was eventually liberated following intensive antihypertensive therapy. A renal biopsy revealed focal segmental glomerulosclerosis (FSGS) with endothelial injury. In addition, the patient had pre-existing, undertreated hypertension. Taken together, the reduced nephron number and impaired vascular development due to LBW may be involved in the development of adult-onset, near fatal hypertensive emergency and subsequent AKI in our case. This case reinforces the long-term cardio-renal risks associated with LBW and illustrates the importance of evaluating perinatal history in the diagnostic workup and successful management of adult-onset nephropathy.

## Introduction

Hypertensive emergencies are characterized by rapid and marked elevation in blood pressure resulting in acute ischemic damage to multiple organs, including the brain, heart, kidneys, and eyes. Renal involvement typically includes thrombotic microangiopathy (TMA)-like lesions and fibrinoid necrosis [1, 2]. Despite advances in antihypertensive therapy, 10–35% of patients with hypertensive emergencies develop long-term dialysis dependence or require kidney transplantation [3], underscoring that renal outcome of hypertensive emergency remains variable.

Low birth weight (LBW; defined as birth weight of 2500 g or less), which is attributed to intrauterine growth restriction (IUGR) and/or preterm birth (birth before 37 weeks of pregnancy), is an important risk factor for the development of various cardiometabolic diseases, such as hypertension, chronic kidney disease (CKD), coronary heart disease, and type 2 diabetes in adulthood [4–9]. In addition, both a systematic review of observational studies [10] and a nationwide Norwegian birth cohort study [11] have suggested that LBW is associated with a higher incidence of end-stage kidney disease (ESKD). It has been postulated that LBW-associated reduction in nephron endowment results in compensatory hyperfiltration and increased intraglomerular pressure, contributing to the development of glomerular hypertrophy, podocyte injury, and ultimately focal segmental glomerulosclerosis (FSGS) in later life [7, 12]. Here, we report a rare case of hypertensive emergency with biopsy-proven FSGS in a patient who had a history of very low birth weight (VLBW).

## Case report

**Fig. 1:**
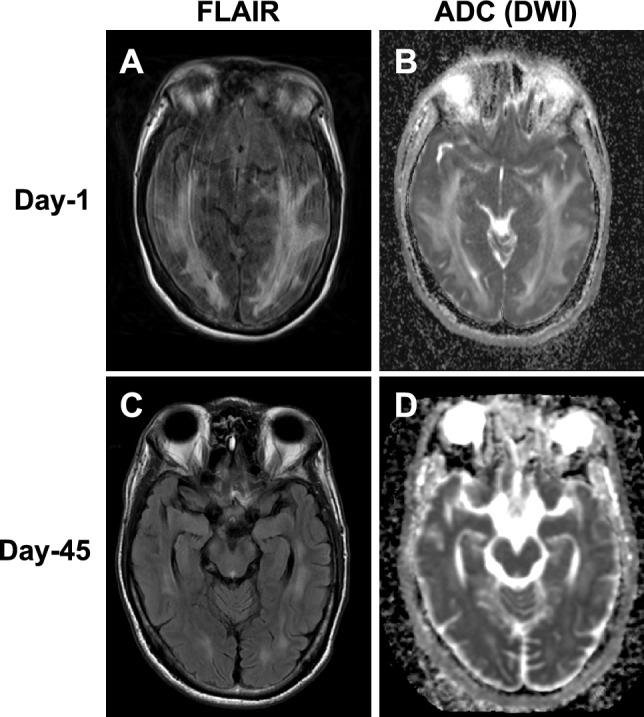
Brain magnetic resonance imaging scan. **A** The fluid attenuated inversion recovery (FLAIR) images showed high-intensity zones in the bilateral occipital lobes on the 1st hospital day. **B** Diffusion-weighted imaging (DWI) scans revealed increased apparent diffusion coefficient (ADC) These abnormalities were resolved on the 45th hospital day (**C** and **D**)

A forty-year-old man, who was born prematurely at 32 weeks of gestation with a birth weight of 1300 g, presented with hypertensive emergency. His past medical history involved a traumatic frontal lobe injury. He had no family history suggestive of hereditary kidney disease. A routine health examination revealed a serum creatinine (sCr) of 0.81 mg/dL, proteinuria ( ±), and high blood pressure (181/125 mmHg) 15 months before admission. Although the patient was advised to seek medical attention for further examination, the patient did not follow the recommendation and hypertension had been left untreated. One year later, he developed worsening kidney function (sCr = 1.41 mg/dL, proteinuria (3 +)) and hypertension (222/142 mmHg). He was presented to an outside hospital with dizziness, headache, elevated blood pressure (233/142 mmHg), and cerebral edema detected by the brain computed tomography (CT). The patient was immediately transferred to our hospital following the initiation of intravenous nicardipine administration. Upon admission, the patient was alert (Glasgow Coma Scale (GCS) score, 15) but exhibited mild disorientation. His blood pressure remained elevated at 199/120 mmHg, despite a continuous intravenous infusion of nicardipine. Laboratory findings revealed normocytic anemia with schistocytes, thrombocytopenia (platelet count, 76 × 10^3^/μL), an elevated lactate dehydrogenase (LDH) level (816 U/L), and undetectable haptoglobin, indicative of microangiopathic hemolytic anemia (MAHA). ADAMTS13 activity was preserved at 97%. In addition, he had severe renal impairment, manifested by an elevated serum creatinine concentration (7.32 mg/dL), marked proteinuria (protein-to-creatinine ratio of 2.17 g/gCre), and hematuria. Hypokalemia (3.2 mmol/L) and marked increases in plasma renin activity (102 ng/mL/h) and plasma aldosterone concentration (179 pg/mL) were detected in the patient. Brain magnetic resonance imaging (MRI) revealed high signal intensity on fluid-attenuated inversion recovery (FLAIR) images in the bilateral parieto-occipital lobes, indicating the development of posterior reversible encephalopathy syndrome (PRES) (Fig. 1).

**Fig. 2:**
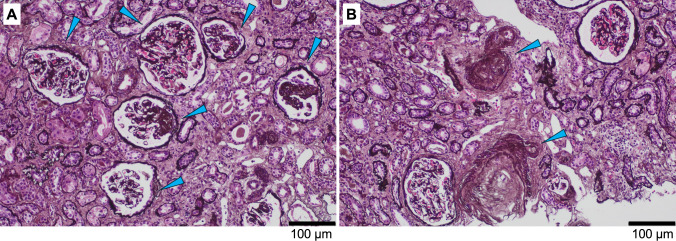
Light microscopy of the kidney biopsy Global/segmental glomerular sclerosis in the glomeruli (**A**) and an onion skin-like lesion in the small renal arteries (**B**) were detected on Periodic Acid Methenamine silver (PAM) staining

A renal biopsy was performed on day 37 after successful management of blood pressure and recovery of renal function (Fig. 2). In the specimens from the kidney, 54 glomeruli were identified. One glomerulus showed global sclerosis, and five showed segmental sclerosis on Periodic Acid Methenamine silver (PAM) staining (Fig. 2A). These segmental lesions were classified as the not otherwise specified (NOS) variant. There was an onion skin-like lesion of small renal arteries (Fig. 2B). Immunofluorescence studies were overall negative. Taken together, the histopathological findings were consistent with FSGS and endothelial damage reflecting the acute pathological changes induced by hypertensive emergency.

**Fig. 3:**
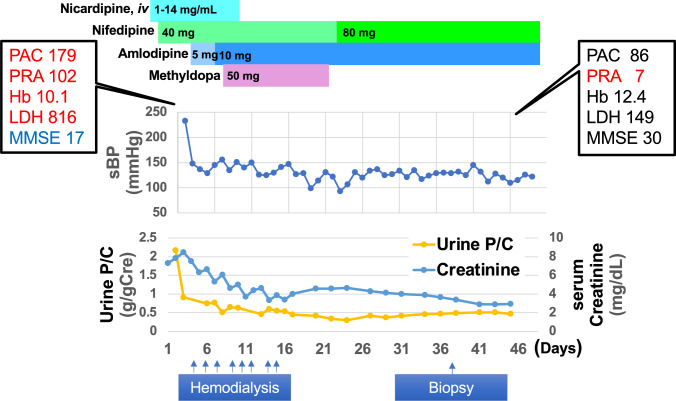
Clinical course PAC; Plasma aldosterone concentration (pg/mL), PRA; Plasma renin activity (ng/mL/hr), Hb; Hemoglobin (g/dL), LDH; Lactate dehydrogenase (U/L), MMSE; Mini mental state examination

The clinical course of the patient is summarized in Fig. 3. Initial management involved a continuous intravenous nicardipine infusion (1–14 mg/h). Following blood pressure stabilization, he was transitioned to oral antihypertensive agents, including nifedipine, amlodipine, and methyldopa [13]. His sCr peaked at 8.47 mg/dL on day 3, necessitating hemodialysis. His renal function gradually recovered and hemodialysis was discontinued on day 15. In addition, the laboratory markers of microangiopathic hemolytic anemia, including thrombocytopenia and elevated LDH, were resolved. The Mini Mental State Examination (MMSE) score was also improved from 17 to 30 and a follow-up brain MRI on day 45 demonstrated resolution of the cerebral edema (Fig. 1). On day 49, he was transferred to a rehabilitation facility. Losartan (25 mg/day) was initiated in the outpatient setting after the stabilization of renal function.

## Discussion

We herein report the rare case of a patient with a history of very low birth weight (VLBW; defined as birth weight of 1500 g or less) who required hemodialysis due to acute kidney injury (AKI) associated with hypertensive emergency. Despite poor prognostic factors including severe hypertension [2], renal dysfunction requiring temporary hemodialysis, proteinuria [14], and evidence of MAHA/TMA [15], the patient achieved substantial renal recovery following successful management of blood pressure. This outcome is consistent with reports showing that renal function can be reversible in severe hypertensive emergencies with prompt intervention [2, 16]. The hyperactivation of renin–angiotensin–aldosterone system (RAAS), evidenced by marked increases in plasma PRA and aldosterone concentration, likely contributed to both the severity of the hypertension and intrarenal hemodynamic stress [17, 18]. In our case, RAAS inhibitor therapy was initiated after the stabilization of renal function but not during the acute phase since the clinical benefits induced by RAAS inhibitors in the patients with AKI were under debate [19, 20]. However, earlier initiation of RAAS inhibitor would have been more appropriate since data obtained from the recent studies conducted in the patients with hypertensive emergency suggest that earlier initiation of RAAS inhibitor improves renal outcomes regardless of the baseline glomerular filtration rate (GFR) values [21, 22].

**Fig. 4:**
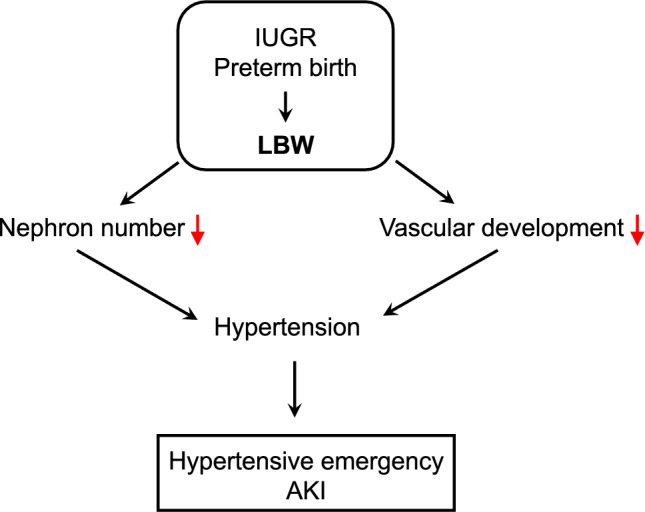
Potential mechanism explaining the relationship between LBW and hypertensive emergency IUGR; intrauterine growth restriction, LBW; low birth weight, AKI; acute kidney injury

Several reports have described young adults who developed hypertensive emergency and FSGS with varying degrees of AKI [16, 18, 23]. The FSGS lesion observed in our patient primarily reflects the acute pathological changes induced by hypertensive emergency. To our best knowledge, this is the first report of hypertensive emergency and subsequent FSGS and AKI who had a documented history of VLBW. LBW is associated with an unfavorable clinical outcome in various renal diseases including FSGS, IgA nephropathy, minimal change disease, and idiopathic membranous nephropathy [24–27]. The relationship between LBW and negative renal outcomes could be explained by the reduced nephron number and subsequent renal damage in adulthood. In our case, undertreated, pre-existing hypertension could contribute to the development of hypertensive emergency [28]. Indeed, a low number of nephrons is associated with the development of hypertension in later life [5, 29]. In addition, IUGR and preterm birth negatively impacts vascular development and thus impairs vascular structure and function. Indeed, previous studies suggest that people with a history of LBW have reduced capillary density and arteriolar narrowing, likely resulting in increases in peripheral vascular resistance and blood pressure [30, 31]. Taken together, these findings suggest that reduced nephron number and defective vascular development associated with LBW could be involved in the development of adult-onset, near fatal hypertensive emergency and subsequent AKI in our patient (Fig. 4).

In conclusion, this case supports the hypothesis that LBW and presumed congenital nephron deficit and defective vascular development can predispose individuals to hypertensive emergency and AKI later in life. In Japan, the rate of LBW has been increasing[9] and clinicians should evaluate perinatal history, including birth weight, when examining the patient with nephropathy. In addition, long-term surveillance from a young age is essential for the early detection and prompt treatment of hypertension and renal dysfunction since LBW is associated with an unfavorable clinical outcome in renal diseases [24–27]. Although our patient was diagnosed as having hypertension at the checkup and recommended for medical examination 15 months before admission, he did not seek medical care, which was considered to be a cause of the development of hypertensive emergency and AKI.

## References

[1] Rubin S, Cremer A, Boulestreau R, Rigothier C, Kuntz S, Gosse P. Malignant hypertension: diagnosis, treatment and prognosis with experience from the Bordeaux cohort. J Hypertens. 2019;37(2):316–24. 10.1097/HJH.0000000000001913. 30160657 10.1097/HJH.0000000000001913

[2] Amraoui F, Bos S, Vogt L, van den Born BJ. Long-term renal outcome in patients with malignant hypertension: A retrospective cohort study. BMC Nephrol. 2012;13:71. 10.1186/1471-2369-13-71. 22846257 10.1186/1471-2369-13-71 PMC3470982

[3] Lip GY, Beevers M, Beevers DG. Complications and survival of 315 patients with malignant-phase hypertension. J Hypertens. 1995;13(8):915–24. 10.1097/00004872-199508000-00013. 8557970 10.1097/00004872-199508000-00013

[4] Barker DJ, Osmond C, Golding J, Kuh D, Wadsworth ME. Growth in utero, blood pressure in childhood and adult life, and mortality from cardiovascular disease. BMJ. 1989;298(6673):564–7. 10.1136/bmj.298.6673.564. 2495113 10.1136/bmj.298.6673.564 PMC1835925

[5] Schreuder MF, Nauta J. Prenatal programming of nephron number and blood pressure. Kidney Int. 2007;72(3):265–8. 10.1038/sj.ki.5002307. 17495859 10.1038/sj.ki.5002307

[6] Bertram JF, Douglas-Denton RN, Diouf B, Hughson MD, Hoy WE. Human nephron number: Implications for health and disease. Pediatr Nephrol. 2011;26(9):1529–33. 10.1007/s00467-011-1843-8. 21604189 10.1007/s00467-011-1843-8

[7] Kanda T, Murai-Takeda A, Kawabe H, Itoh H. Low birth weight trends: Possible impacts on the prevalences of hypertension and chronic kidney disease. Hypertens Res. 2020;43(9):859–68. 10.1038/s41440-020-0451-z. 32393862 10.1038/s41440-020-0451-z

[8] Murai-Takeda A, Kanda T, Azegami T, Hirose H, Inokuchi M, Tokuyama H, et al. Low birth weight is associated with decline in renal function in Japanese male and female adolescents. Clin Exp Nephrol. 2019;23(12):1364–72. 10.1007/s10157-019-01784-9. 31494799 10.1007/s10157-019-01784-9

[9] Kanda T, Takeda A, Hirose H, Abe T, Urai H, Inokuchi M, et al. Temporal trends in renal function and birthweight in Japanese adolescent males (1998-2015). Nephrol Dial Transplant. 2018;33(2):304–10. 10.1093/ndt/gfw428. 28339560 10.1093/ndt/gfw428 PMC5837670

[10] White SL, Perkovic V, Cass A, Chang CL, Poulter NR, Spector T, et al. Is low birth weight an antecedent of CKD in later life. A systematic review of observational studies. Am J Kidney Dis. 2009;54(2):248–61. 10.1053/j.ajkd.2008.12.042. 19339091 10.1053/j.ajkd.2008.12.042

[11] Vikse BE, Irgens LM, Leivestad T, Hallan S, Iversen BM. Low birth weight increases risk for end-stage renal disease. J Am Soc Nephrol. 2008;19(1):151–7. 10.1681/ASN.2007020252. 18057216 10.1681/ASN.2007020252 PMC2391041

[12] Fogo AB. Causes and pathogenesis of focal segmental glomerulosclerosis. Nat Rev Nephrol. 2015;11(2):76–87. 10.1038/nrneph.2014.216. 25447132 10.1038/nrneph.2014.216 PMC4772430

[13] Gosse P, Boulestreau R, Brockers C, Puel C, Rubin S, Cremer A. The pharmacological management of malignant hypertension. J Hypertens. 2020;38(11):2325–30. 10.1097/HJH.0000000000002547. 32649635 10.1097/HJH.0000000000002547

[14] González R, Morales E, Segura J, Ruilope LM, Praga M. Long-term renal survival in malignant hypertension. Nephrol Dial Transplant. 2010;25(10):3266–72. 10.1093/ndt/gfq143. 20299339 10.1093/ndt/gfq143

[15] van den Born BJ, Honnebier UP, Koopmans RP, van Montfrans GA. Microangiopathic hemolysis and renal failure in malignant hypertension. Hypertension. 2005;45(2):246–51. 10.1161/01.HYP.0000151620.17905.ee. 15596574 10.1161/01.HYP.0000151620.17905.ee

[16] Fukuda K, Shimizu A, Kaneko T, Masuda Y, Yasuda F, Fukui M, et al. A case of secondary focal segmental glomerulosclerosis associated with malignant hypertension. CEN Case Reports. 2013;2(1):68–75. 10.1007/s13730-012-0041-2PMID-28509227PMC-PMC5413729. 28509227 10.1007/s13730-012-0041-2 PMC5413729

[17] Zhang B, Xing C, Yu X, Sun B, Zhao X, Qian J. Renal thrombotic microangiopathies induced by severe hypertension. Hypertens Res. 2008;31(3):479–83. 10.1291/hypres.31.479. 18497467 10.1291/hypres.31.479

[18] Chowdhary M, Kabbani AA, Tobey D, Hope TD. Posterior reversible encephalopathy syndrome in a woman with focal segmental glomerulosclerosis. Neuropsychiatr Dis Treat. 2015;11:1111–4. 10.2147/NDT.S84010. 25960654 10.2147/NDT.S84010 PMC4411014

[19] Brar S, Ye F, James MT, Hemmelgarn B, Klarenbach S, Pannu N. Association of angiotensin-converting enzyme inhibitor or Angiotensin receptor blocker use with outcomes after acute kidney injury. JAMA Intern Med. 2018;178(12):1681–90. 10.1001/jamainternmed.2018.4749. 30422153 10.1001/jamainternmed.2018.4749 PMC6583606

[20] Feidakis A, Panagiotou MR, Tsoukakis E, Bacharaki D, Gounari P, Nikolopoulos P, et al. Impact of angiotensin-converting enzyme inhibitors or angiotensin receptor blockers on acute kidney injury in emergency medical admissions. J Clin Med. 2021. 10.3390/jcm10030412. 33499035 10.3390/jcm10030412 PMC7865425

[21] Endo K, Hayashi K, Hara Y, Miyake A, Takano K, Horikawa T, et al. Impact of early initiation of renin-angiotensin blockade on renal function and clinical outcomes in patients with hypertensive emergency: A retrospective cohort study. BMC Nephrol. 2023;24(1):68. 10.1186/s12882-023-03117-1. 36949416 10.1186/s12882-023-03117-1 PMC10035153

[22] Ueno M, Fujii W, Ono W, Murata H, Fujigaki Y, Shibata S. Renin inhibition and the long-term renal function in patients with hypertensive emergency: A retrospective cohort study. Am J Hypertens. 2024;37(6):407–14. 10.1093/ajh/hpad099. 37819695 10.1093/ajh/hpad099

[23] Abdullah HM, Ullah W, Ahmad E, Anwer F. Posterior reversible encephalopathy syndrome in malignant hypertension secondary to focal segmental glomerulosclerosis. BMJ Case Rep. 2016;2016:bcr2016216512. 10.1136/bcr-2016-216512. 27535734 10.1136/bcr-2016-216512 PMC5015126

[24] Hodgin JB, Rasoulpour M, Markowitz GS, D’Agati VD. Very low birth weight is a risk factor for secondary focal segmental glomerulosclerosis. Clin J Am Soc Nephrol. 2009. 10.2215/CJN.01700408. 19019999 10.2215/CJN.01700408 PMC2615706

[25] Duncan RC, Bass PS, Garrett PJ, Dathan JR. Weight at birth and other factors influencing progression of idiopathic membranous nephropathy. Nephrol Dial Transplant. 1994. 10.1093/ndt/9.7.875a. 7970135

[26] Zidar N, Čavić MA, Kenda RB, Koselj M, Ferluga D. Effect of intrauterine growth retardation on the clinical course and prognosis of IgA glomerulonephritis in children. Nephron. 1998. 10.1159/000044987. 9609458 10.1159/000044987

[27] Zidar N, Čavić MA, Kenda RB, Ferluga D. Unfavorable course of minimal change nephrotic syndrome in children with intrauterine growth retardation. Kidney Int. 1998. 10.1046/j.1523-1755.1998.00121.x. 9767550 10.1046/j.1523-1755.1998.00121.x

[28] Vaughan CJ, Delanty N. Hypertensive emergencies. Lancet. 2000. 10.1016/S0140-6736(00)02539-3. 10972386 10.1016/S0140-6736(00)02539-3

[29] Keller G, Zimmer G, Mall G, Ritz E, Amann K. Nephron number in patients with primary hypertension. N Engl J Med. 2003;348(2):101–8. 10.1056/NEJMoa020549. 12519920 10.1056/NEJMoa020549

[30] Kistner A, Jacobson L, Jacobson SH, Svensson E, Hellström A, Kistner A. Low gestational age associated with abnormal retinal vascularization and increased blood pressure in adult women. Pediatr Res. 2002;51(6):6. 10.1203/00006450-200206000-00003. 10.1203/00006450-200206000-00003 12032260

[31] Mitchell P, Liew G, Rochtchina E, Wang JJ, Robaei D, Cheung N, et al. Evidence of arteriolar narrowing in low-birth-weight children. Circulation. 2008. 10.1161/CIRCULATIONAHA.107.747329. 18625895 10.1161/CIRCULATIONAHA.107.747329

